# A Reverse Transcriptase-Dependent Mechanism Is Essential for Murine Preimplantation Development

**DOI:** 10.3390/genes2020360

**Published:** 2011-05-18

**Authors:** Ilaria Sciamanna, Patrizia Vitullo, Angela Curatolo, Corrado Spadafora

**Affiliations:** Italian National Institute of Health (ISS), Viale Regina Elena 299, 00161 Rome, Italy; E-Mails: ilaria.sciamanna@iss.it (I.S.); patrizia.vitullo@iss.it (P.V.); angela.curatolo@iss.it (A.C.)

**Keywords:** preimplantation embryo development, retrotransposon, LINE-1, reverse transcriptase inhibitors, tumorigenesis

## Abstract

LINE-1 (Long Interspersed Nuclear elements) and HERVs (Human Endogenous Retroviruses) are two families of retrotransposons which together account for about 28% of the human genome. Genes harbored within LINE-1 and HERV retrotransposons, particularly that encoding the reverse transcriptase (RT) enzyme, are generally expressed at low levels in differentiated cells, but their expression is up-regulated in embryonic tissues and transformed cells. Here we review evidence indicating that the LINE-1-encoded RT plays regulatory roles in early embryonic development. Indeed, antisense-mediated inhibition of expression of a highly expressed LINE-1 family in mouse zygotes caused developmental arrest at the two- or four-cell embryo stages. Development is also arrested when the embryo endogenous RT activity is pharmacologically inhibited by nevirapine, an RT inhibitor currently employed in AIDS treatment. The arrest of embryonic development is irreversible even after RT inhibition is removed and it is associated with subverted gene expression profiles. These data indicate an early requirement for LINE-1-encoded RT to support early developmental progression. Consistent with this, recent findings indicate that a reverse transcription wave is triggered in the zygote a few hours after fertilization and is propagated at least through the first two rounds of cell division. On the whole these findings suggest that reverse transcription is strictly required in early embryos as a key component of a novel RT-dependent mechanism that regulated the proper unfolding of the developmental program.

## 1. A Highly Dynamic Genomic Landscape in Preimplantation Embryos

The earliest stages of embryogenesis, from fertilization to the second cell division, identify a crucial window during which many essential genome-wide events occur simultaneously and shape the subsequent developmental program. Several genetic and epigenetic events are activated in this developmental frame, e.g., the fusion of paternal and maternal genomes, genome demethylation, chromatin remodeling in pronuclei, activation of embryonic gene expression and genome replication. These events do not only affect the earliest stages of development, but they activate the molecular machinery responsible for early blastomere commitment with far-reaching implications for development of the growing embryo. This sets the structural and functional foundations upon which the subsequent developmental program will build up. In the mouse embryo totipotency wanes during early cell divisions: the blastomeres from two-cell embryos are fully totipotent and each one can support the development of a complete individual. After the next cell division, individual four-cell blastomeres originate miniature blastocysts. After yet another round, individual eight-cell blastomeres produce trophoblastic vesicles [1]. Thus, early blastomeres undergo changes that restrict their potential. This indicates that the molecular process leading to the first manifestation of cell specification, *i.e.*, the distinction between the Inner Cell Mass (ICM) and Trophoblast (TE), is already at work in the first few rounds of cell division [2].

Among events typically occurring in this window, genome-wide DNA demethylation is triggered soon after fertilization in the zygote pronuclei [3]. It involves the genomes of both gametes, yet proceeds in sharply different ways in the two pronuclei: the paternal genome undergoes fast massive demethylation, whereas the maternal genome is demethylated through a passive, slowly-progressing DNA replication-dependent mechanism [3,4]. Both genomes are re-methylated around the time of implantation to different extents in embryonic and extraembryonic lineages. The two genomes therefore behave initially as distinct and independent entities in spite of their being both contained within the same zygote. This conclusion is consistent with the surprising finding that the parental genomes, though being packed in the same nuclei of early mouse embryos, remain topologically separated for some time: their separation is preserved up to the four-cell stage, then gradually disappears [5,6].

Parallel to DNA demethylation, genome-wide conformational changes take place at the chromatin level. These changes dramatically reset the genome organization during the process that converts gametic nuclei into zygotic pronuclei. The paternal genome undergoes extensive chromatin reorganization, in which histones replace protamines and the highly compact sperm chromatin is converted to a flexible nucleosomal conformation suitable to support genomic functions [3,7]. Chromatin reorganization actually continues as an ongoing process throughout preimplantation development and correlates with cell lineage commitment and loss of pluripotency [8]. Both DNA demethylation and chromatin remodeling have critical importance: they allow the genomes of differentiated gametes to first return to full totipotency [3], and later restrict the totipotency of early blastomeres to support cell fate determination.

In consequence of these epigenetic and conformational changes both parental genomes, within only a few hours after fertilization, acquire the competence to replicate and activate embryonic gene expression under zygotic control, which replaces the maternally inherited control at fertilization. The activation of embryonic gene expression is a well-regulated phenomenon that follows a typical wave-like pattern and leads to the progressive activation of discrete sets of genes. At least two major and two minor activation events take place sequentially. The first major wave leads to the zygotic genome activation (ZGA), which peaks at the two- to four-cell embryo stages (oocyte-to-embryo transition). The second major wave follows: it peaks at the eight-cell embryo stage (mid-preimplantation gene activation, MGA) and contributes to differentiation changes in late preimplantation. The third and fourth waves peak at the morula and blastocyst stages, respectively [9–11]. The molecular mechanisms underlying these processes are largely obscure: both the basis of their timing and their mutual links remain to be clarified in this intricate program.

## 2. The Emergence of Retrotransposition in Developmental Control

In recent years intriguing evidence have accumulated, which have delineated an unexpected side of developmental control and have pinpointed new roles of retroelements in this control. Retroelements (or retrotransposons) are abundant components of the genome of higher metazoa. They are classified in three major groups: LINE-1 (Long Interspersed Nuclear Elements-1), Alu/SINE (Short Interspersed Nuclear Elements) and ERVs (Endogenous Retroviruses), which together account for about 45% of the human genome [12,13]. LINE-1 make up the largest retrotransposon family, accounting alone for 17% of the human genome. The majority of LINE-1 family members is represented by truncated elements unable to retrotranspose, but about one hundred of LINE-1 elements are full-length and retrotransposition-competent [14,15]. Full-length LINE-1 and ERV elements harbor genes coding for reverse transcriptase (RT) enzymes, which make them capable of autonomous retrotransposition. Alu/SINEs—which lack the RT-coding gene—instead exploit the retrotransposition machinery provided by LINE-1 [15]. The RT plays an essential catalytic role in retrotransposition and determines the successful spreading of retrotransposon copies that colonize the genomes.

Due to the apparent lack of any obvious role, retroelements were traditionally regarded as parasitic elements [16,17], collectively composing a genome portion defined as “junk DNA”. Growing evidence, however, have progressively challenged that view and indicate that in fact retroelements affect the host cell transcriptome. A detailed review of the different molecular mechanisms through which retroelements affect host gene expression would be out of scope in this article, but many excellent reviews cover the subject in depth [15,18–20].

Here we focus on findings that delineate a functional link between retroelement/retrotransposon activity and early embryo development. Studies of retrotransposon expression show that a significant proportion of all protein-coding cDNA sequences (about 13% in mouse oocytes and 7.5% in two-cell stage embryos) contain retrotransposon-derived sequences, the expression of which is developmentally regulated [21,22]. During their program of developmentally regulated expression, retroelements have been suggested to provide “alternative” promoters capable of regulating transcription of host genes in early cleavage-stage embryos [23]. It is worth stressing that this occurs concomitant with the overall demethylation of the embryonic genome [4]. These findings contrast with the early proposal that retroelements are non-functional parasitic sequences and suggest instead their implication in genome-wide regulatory mechanisms operating in early embryogenesis. Furthermore, the potential of retroelements to regulate not only coding genes, but also non-coding sequences, is clearly emerging. Indeed, a major reprogramming of small RNA expression profiles occurs in the earliest developmental phases, with a transition from retrotransposon-derived small interfering (si) and *piwi* (pi) RNAs to zygotically synthesized micro RNAs (miRNAs) [24]. Some of the siRNAs and piRNAs are transiently up-regulated and directed against specific retrotransposon classes: this induces drastic changes in the profile of endogenous small RNAs, associated with the transition from oocyte to embryo. Furthermore, growing data point out that preimplantation embryos offer a highly permissive environment for precocious retroposon expression [25–27] and for retrotransposition events. Indeed, most LINE-1 retrotransposition events are demonstrated to take place in human and mouse early embryos [28–30]. Retrotransposon activities are dramatically down-regulated in differentiated tissues [31]. These findings depict a temporal concurrence between DNA demethylation and retrotransposon expression, with possible consequences on the establishment of small RNA profiles in early embryo cell divisions, in the timeframe during which blastomeres progressively loose totipotency and diversify. The evidence for retrotransposition occurrence in early embryos oppose models that recognized the germ line as the preferential or exclusive site of retrotransposition [32].

The activation of retroelements does not generate an uncontrolled burst of expression and/or retrotransposition in the embryo. On the contrary, it is a well-modulated developmental phenomenon, accurately regulated by various integrating mechanisms, including differential methylation [33–35], endogenous RNA interference (RNAi) [36,24] and interactions among regulatory proteins [37,38]. In this framework, retrotransposons, and the genes harbored therein, emerge as new potential players in regulatory networks modulating genomic functions in early development. What follows is an account of studies carried out in our laboratory to clarify these mechanisms.

## 3. An Endogenous RT Activity Operates in Spermatozoa and Early Embryos

An early hint that struck our imagination and first suggested a role for retrotransposon-harbored genes in embryo development was the unexpected finding that an endogenous RT activity is present in murine spermatozoa [39]. That finding stemmed out from studies in which we had characterized a peculiar chromatin fraction in mouse spermatozoa, that: (i) retains a nucleohistone structure within the nucleoprotamine bulk; (ii) is organized in nucleosomes; (iii) is conformationally “accessible” as revealed by nuclease sensitivity, and, most importantly; (iv) is enriched in sequences of retrotransposon origin [40]. That fraction was found to be undermethylated [41], raising the possibility that retrotransposons were expressed during spermatogenesis and coded for products that were stored in mature gametes. That hypothesis proved true: we found that the sperm RT, far from being a non-functional remnant from fossil genetic elements, is indeed a biologically active enzyme able to reverse-transcribe cDNA copies from exogenous RNA molecules incubated with spermatozoa [39]. The newly generated cDNAs in sperm cells can be delivered to oocytes and propagated in embryos by simply using a sperm/RNA mixture, instead of pure sperm cells, in *in vitro* fertilization (IVF) assays. These results provided the foundation for the phenomenon called Sperm Mediated “Reverse” Gene Transfer (SMRGT), in which new phenotypic traits can be generated in animals starting from RNA templates, as described elsewhere [42–46].

We further assessed that an RT activity is also abundant in early embryos. That was ascertained by adapting an RT-PCR-based assay using the MS2 phage genomic RNA as a pure RNA template and zygote- or embryo (two-cell or four-cell stage) lysate as the sole source of RT activity [47]. Those assays yielded retro-transcribed cDNA products, demonstrating that an RT activity is relatively abundant and biologically effective in early blastomeres.

*A priori* the embryonic RT can be of two possible sources: (i) autonomously synthesized in early embryos via transcription of LINE-1 and ERV families, whose expression is reactivated concomitant with genome demethylation (see above); (ii) non-mutually exclusively, RT may be specifically carried over by spermatozoa at fertilization. It is worth recalling in this respect that fertilizing spermatozoa contribute not only their own nucleus to oocytes, but also deliver a variety of macromolecules presumed to be of functional relevance in early embryogenesis [48–50]. In recent work, currently in progress, we have found that sperm cells indeed deliver functional RT to oocytes at fertilization, which has a precocious role in the zygote, as revealed by the occurrence of reverse transcription enzymatic activity predominantly in the male pronucleus. Thus, the RT of sperm cell origin is part of the set of activities expressed in early development.

## 4. Inhibition of Endogenous RT Arrests Development

**Figure 1:**
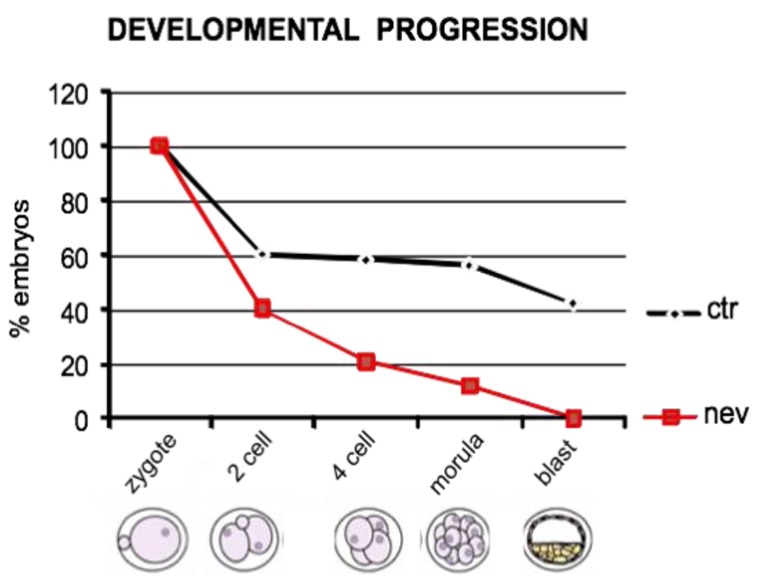
Nevirapine treatment arrests early mouse embryo development. The graphs represent the percentage of viable mouse embryos obtained from *in vitro* fertilization experiments in ordinary culture conditions (controls, ctr, black line) or in the presence of nevirapine (nev, red line), at each developmental stage (Modified from Pittoggi *et al.*, 2003).

At this point it was important to clarify whether the embryonic RT has roles in development or whether it is a mere non-functional evolutionary remnant. To address that question, we designed RT inhibition experiments using two independent approaches. In the first one, preimplantation embryos were exposed to nevirapine, a non-nucleosidic RT inhibitor employed in AIDS treatment [51]. The drug was added to the embryo culture medium and developmental progression was monitored [47]. When embryos were exposed to nevirapine at as early a stage as the zygote, development arrested irreversibly at the two- or four-cell stages; none of the RT-inhibited embryos reached the blastocyst stage (summarized in Figure 1). Developmental arrest was also observed when two- or four-cell stage embryos were exposed to the drug. The embryo developmental arrest was associated with a significant drop in endogenous RT activity, as determined in PCR-based assays. The RT-inhibitory treatment however had no consequence when administered either within the first five hours post-insemination or from the eight-cell stage onwards. These data suggest that: (i) the zygotic and embryonic RT activity is essential for early embryogenesis, and (ii) the sensitivity to RT inhibition is restricted to a window between the late one- and the four-cell stages. Interestingly, that window temporally overlaps with the first major wave of embryonic gene activation [9–11]. We therefore analyzed a panel of “housekeeping” and developmentally modulated genes: nevirapine induced profound alterations of gene expression profiles in arrested compared to control embryos. These data suggest that the endogenous RT is part of a gene expression regulatory mechanism in early embryos [47].

**Figure 2:**
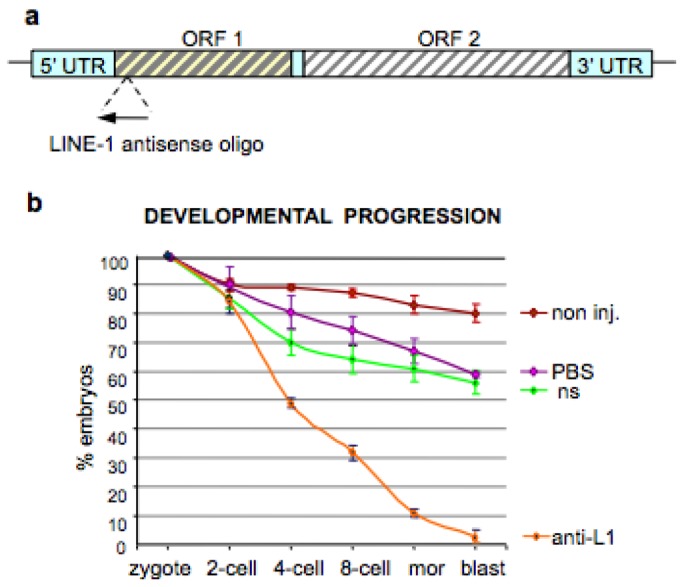

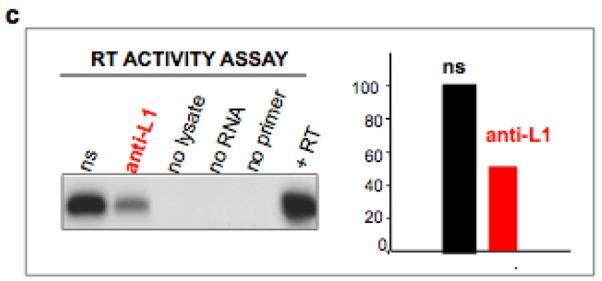
Microinjection of LINE-1-specific antisense oligonucleotides arrests embryo development at preimplantation stages. (**a**) Map of a murine full-length LINE-1/L1 element. The position of LINE-1 antisense oligonucleotide used by Beraldi *et al.* (2006) is symbolized by the arrow near the 5′ UTR; (**b**) Developmental progression in groups of mouse embryos treated as follows: non-injected (non inj, brown line), or injected with PBS (purple line), nonsense oligonucleotide (NS, green line) and L1 antisense oligonucleotide (anti-L1, orange line). Data represent mean values, and bars the standard deviation, from two to five experiments; (**c**) Functional assay of the endogenous RT activity (MS2 phage RNA was used as template and embryo lysates as the RT source). Lysates were either from nonsense oligonucleotide-injected embryos (ns), or from arrested embryos injected with L1 antisense (anti-L1). Negative control reactions included no lysate, no RNA or no primers, while positive controls utilized commercial RT. Retrotranscribed MS2 cDNA products were quantified by densitometry. Mean and standard deviations were calculated from three experiments and are expressed relative to values obtained in positive controls (taken as 100%) (Modified from Beraldi *et al.*, 2006).

To substantiate these results and rule out possible artifacts caused by non-specific off-target effects of nevirapine, we designed a second set of experiments to specifically down-regulate a highly expressed LINE-1 family in murine cells [52]. That LINE-1 family is regarded as the major, if not unique, source of RT activity responsible for most retrotransposition events in murine cells. We used an antisense oligonucleotide targeting the 5′-end of ORF1 in the murine LINE-1/L1 element (Figure 2A). LINE-1 antisense oligonucleotide microinjection in zygotes caused a total developmental arrest (Figure 2B), reproducing that induced by nevirapine [53]. Concomitant with this, the endogenous RT activity was again significantly reduced in arrested compared to control embryos at the same stage (Figure 2C). In contrast, microinjection of non-specific oligonucleotide of same length but scrambled sequence had no developmental consequence. These results provide a proof-of-principle that the endogenous RT activity is required for progression of early cleavage embryos and identify an active LINE-1 family as a major source of RT activity. In synthesis, therefore, both the sperm-derived and the embryo newly synthesized RT pools are strictly required for preimplantation development.

The requirement for LINE-1 expression in early embryogenesis is consistent with data implicating an endogenous retrovirus family in early development: indeed, inactivation of murine endogenous retrovirus-L (MuERV-L) by MuERV-L antisense oligonucleotide microinjection into zygotes caused a 30% reduction in the rate of embryonic development [27]. MuERV-L is a member of a precociously expressed retroelement family soon after fertilization; its function is however unclear. Nonetheless, the evidence that its inactivation blocks embryo development—albeit to a milder extent compared to LINE-1—adds weight to the functional role of retroelements in early development.

Recent work, currently in progress in our laboratory, showed that a reverse transcription wave is triggered in mouse zygotes a few hours after fertilization and extends at least up to the two-cell stage. This wave generates cDNA products that are retained in zygotic pronuclei and embryonic nuclei. These products are absent in embryos treated with various RT inhibitors, but not with the DNA polymerase inhibitor aphidicoline, which confirms that they derive from a genuine RT-driven reaction and not from some atypical DNA replication event. To sum up, available evidence thus far highlight a functional link between embryo preimplantation development and the retroposon-encoded RT activity: the latter not only emerges as a distinctive marker of early developmental progression, but also as a necessary component in early embryogenesis. The molecular mechanisms that modulate RT expression in preimplantation embryos are incompletely understood, yet available data consistently indicate a key regulatory role of DNA methylation [34]. Two points are emerging: first, the pattern of methylation of retroelements is regulated by a specific mechanism mediated by small piRNAs [54], and, second, methylation discriminates and differentially targets distinct retroelements families and subfamilies [33,35], and hence contributes to establish their pattern of expression in embryos. It is worth stressing that embryonic development represents the only non-pathological retrotransposition-permissive temporal window in the lifetime of higher organisms.

## 5. The Analogies between Early Embryogenesis and Tumorigenesis

Since the second half of the 19th century, it is a well-established notion that embryos and tumors share common features [55] and that the process of tumor growth reflects, in some cases, the unscheduled reactivation of embryonic programs [56]. That intuition has received ample experimental confirmation from recent findings indicating that typical embryonic genes are re-expressed in cancer cells, including *OCT4* [57], *Homeobox* and *Twist* family members [58–61], as well as several genes acting in organ and tissue ontogenesis [62,63]. Ample array studies of mouse models further document that transcription profiles in certain tumors strikingly recapitulate embryonic developmental patterns [64]. Furthermore, chromosome instability, a hallmark of tumorigenesis, has also been shown to occur commonly in early human embryogenesis [65]. In line with this background, extensive evidence indicate that retrotransposon expression is reactivated in tumorigenic cells and tumor tissues [66–68].

We have directly addressed the implication of endogenous RT in tumorigenesis and tumor cell growth. In parallel with studies of RT in embryogenesis, we investigated human tumorigenic cell lines (melanoma, prostate, colon, thyroid carcinoma and microcitoma). We found that LINE-1-encoded RT is essential to maintain the tumorigenic cells in a highly proliferating, poorly differentiated or de-differentiated state. In contrast, both pharmacological RT inhibitors [69–71] and RNA interference [70,72] against a highly expressed human LINE-1 family [14] reduced cell proliferation, promoted differentiation and, most importantly, limited the tumorigenic potential of the cell lines. These findings opened up the novel therapeutic perspective of using RT inhibitors as potential agents to treat human cancer. Indeed, we have found that RT inhibitors are endowed with powerful anti-cancer activity, both in *in vitro* experiments with tumorigenic cell lines, in *ex vivo* with human leukemia cells [69] and in *in vivo* assays with human cancer cells inoculated in murine models [70]. These results together indicate that a LINE-1-encoded RT-dependent mechanism operates in tumorigenesis, in striking analogy with embryogenesis. This RT-dependent mechanism is crucial in cell fate determination. Its up-modulation by stress, to which retroposons are known to be responsive [73], or, on the contrary, its inhibition (physiological or therapeutically induced), shape the cell potential towards a highly proliferating, poorly differentiated and potentially tumourigenic direction or towards a quiescent and differentiated state.

## 6. A Genome-Wide RT-Dependent Regulatory Mechanism

On the basis of the results discussed here, we propose that LINE-1-encoded RT is a key component of an RT-mediated mechanism that is physiologically triggered at fertilization and remains active for at least the first two cell divisions. The critical importance of the first two cell divisions to support the developmental program of the entire organism has been recalled above. The RT mechanism is then silenced in normal differentiated tissues, but can be erroneously reactivated and give origin to tumors.

In our model, we see LINE-1 elements, possibly in combination with Alu/SINEs and ERVs, as part of regulatory circuits that include specific sets of coding and non-coding sequences. These circuits would constitute an ample genome-wide regulatory network, in which LINE-1 components would exert a controlling role and coding genes would act as downstream targets with regulated expression in embryogenesis and later in adult tissues. The model assumes two main features: (i) LINE-1 are identified as main regulatory elements, and (ii) their regulatory function is exerted through networks able to modulate gene expression. These regulatory circuits operate at the genetic level, via retrotransposition-mediated events in embryos, whereas their functional modulation is determined by epigenetic mechanisms operating throughout the organism's life. An implication is that the LINE-1 elements constituting regulatory circuits must be placed in specific positions relative to other regulatory sequences (miRNAs, ultra-conserved elements) and/or coding genes. Not all LINE-1 copies present in the genome, therefore, necessarily exert regulatory functions. Recent genome-wide studies of LINE-1 distribution in the human genome are consistent with these ideas [74].

The features of the model recall two well-known historical precedents. First, the regulatory functions attributed to and partly demonstrated for retrotransposons in mammalian early development resembles McClintock's early visionary hypothesis of “controlling elements”, *i.e.*, mobile transposons as key modulators of gene expression in plants [75]. As recalled above, this view is now supported by considerable evidence illustrating genetic and epigenetic mechanisms through which retroelements affect the expression of the host genome and hence the cell transcriptome [15,18–20].

Second, LINE-1-dependent regulatory circuits are reminiscent of those hypothesized years ago by Britten and Davidson, who first suggested that repetitive DNA sequences distributed in the genome establish networks endowed with regulatory functions over batteries of coding genes [76,77]. In our view, the model emerging from work discussed in this review fits well with those original groundbreaking ideas and forms a paradigmatic scenario where such ideas can apply. The foundation for a novel view of the genome organization emerges, in which the genome is viewed as a highly dynamic structure, organized in regulatory modules constituted by different integrated components; within this overall organization, mobile elements, genome rearrangements and reshuffling events are constitutive functional features of a mechanism controlling global expression. The central role played by the RT-dependent mechanism in this context fulfills Temin's early prediction that the endogenous RT activity has roles both in normal development and in tumorigenesis [78].
